# Regression-Derived Ileal Endogenous Amino Acid Losses in Broiler Chickens and Cannulated Pigs Fed Corn Fiber, Wheat Bran, and Pectin

**DOI:** 10.3390/ani10112145

**Published:** 2020-11-18

**Authors:** Sunday A. Adedokun, Olayiwola Adeola

**Affiliations:** Department of Animal Science, Purdue University, West Lafayette, IN 47907, USA; ladeola@purdue.edu

**Keywords:** broiler chicken, corn fiber, endogenous amino acid, pectin, pig, wheat bran

## Abstract

**Simple Summary:**

For animal agriculture to be environmentally sustainable, nitrogen excretion must be reduced. This means diet must be formulated to closely meet the requirements of the animal. Accounting for basal endogenous amino acid (EAA) losses during diet formulation helps in formulating diets that closely match amino acid requirements. These studies examined the effect of three or two different ingredient types on basal losses of amino acids in broiler chickens or pigs, respectively. Corn fiber resulted in higher ileal EAA losses compared with wheat bran in broiler chickens. With the exception of His, Leu, and Tyr, there was no difference in basal ileal EAA losses between corn fiber and pectin in cannulated growing pigs. These studies show that in addition to fiber effects, dietary nitrogen sources could have a different effect on basal ileal EAA losses.

**Abstract:**

The objective of these studies was to determine the effect corn fiber (CF), wheat bran (WB) and pectin (PEC) on basal ileal endogenous amino acid (EAA) losses in broiler chickens (Exp. 1) and cannulated pigs (Exp. 2) using the regression method. Semi-purified diets containing 100 g/kg of CF, WB, or PEC (broiler chickens) and CF or PEC (pigs) were fed to replicate cages consisting of eight birds per cage of 21-d-old broiler chickens and six replicates of pigs consisting of one pig per pen. Ileal endogenous His, Glu, and Pro losses were higher (*p* < 0.05) for CF and WB compared with birds fed diets containing PEC. Contrasts between CF and WB showed higher (*p* < 0.05) ileal endogenous nitrogen, total amino acid, His, Ile, Met, Glu, Pro, and Tyr losses in birds fed the CF diets (Exp. 1). Contrasts of EAA losses between birds fed the WB and PEC diets showed higher (*p* < 0.05) losses for His, Glu, and Pro. In the cannulated pigs, CF resulted in higher (*p* < 0.05) ileal endogenous His, Leu, and Tyr losses. In summary, CF induced higher ileal EAA losses in broiler chickens and cannulated pigs.

## 1. Introduction

In the quest to reduce feed cost, improve animal uniformity, and minimize nitrogen excretion into the environment, non-ruminant animal nutritionists have embraced diet formulation on ileal digestible amino acid basis, especially in light of the increasing use of alternative feed ingredients. Diet formulation on a standardized ileal amino acid digestibility (SIAAD) basis is important in diet formulation on apparent digestibility basis because it accounts for basal ileal endogenous amino acid (EAA), hence the use of SIAAD values is more attractive. The relative advantages of diet formulation on SIAAD basis have been previously discussed [1,2,3,4,5].

Several factors, including dietary protein and fiber levels, could influence ileal EAA losses in non-ruminant animals [1,4]. Dietary fiber contributes to an increase in EAA losses because of its effects on mucous secretion and cell proliferation [6]. In order to obtain consistent and reliable EAA losses data for standardization of apparent ileal amino acid digestibility values, it is important to evaluate the effect of different dietary components, such as fiber type (soluble vs. non-soluble) and viscosity, of feed ingredients on basal ileal EAA flow. For instance, high levels of dextrose compared with high levels of cornstarch in a typical nitrogen-free diet (NFD) resulted in significantly higher ileal EAA losses in broiler chickens [7]. Similarly, ileal EAA losses in mature broiler chickens were significantly higher in birds fed an NFD with a high dietary electrolyte balance (108 vs. 219 mEq/kg, [8]). Additionally, the influence of fiber [9] and fiber levels in healthy and challenged broiler chickens [10], phytase and phytic acid [11,12,13] have been reported. The method of estimating basal ileal EAA losses is also important. The total basal ileal EAA losses obtained by the regression method was higher in younger broiler chickens compared with values from the NFD method (day 5; 11,492 vs. 8692 mg/kg DM intake [14]); however, this difference disappeared by day 15 [14]. It has been reported [12] that there was minimal difference in basal ileal EAA losses obtained through NFD, low casein diet, and regression methods in pigs.

The importance of estimating EAA losses and the effects of different dietary components on EAA losses in poultry is well documented [3,4,9,14,15,16,17]. Despite the availability of information on the role of dietary fiber on ileal EAA losses, the effect of the inherent characteristics of the different components of feed ingredients using the regression methods has not been extensively studied in broiler chickens.

Dietary fiber affects EAA losses in a variety of ways. First, highly soluble fiber with high water-holding capacity (WHC) may result in high ileal EAA losses as a result of its effect on the epithelial wall of the gastrointestinal tract (GIT). Secondly, highly soluble fiber reduced feed intake in pigs [18], consequently resulting in a higher proportion of amino acids of endogenous origin in the digesta. However, high solubility does not always translate to high WHC [19]) and EAA flow changes with the level of fiber inclusion in the diet. An increase in EAA flow in pigs fed a NFD with an increase in dietary fiber was reported [20], whereas a high level of solkafloc (75 vs. 25 g/kg diet) in the NFD did not significantly influence basal ileal EAA flow in 26-d old broiler chickens [10]. Although it has been reported that different fiber sources do influence ileal EAA flow, there is little information on the effect of different fiber sources on ileal EAA flow in broiler chickens using the regression method.

Despite the plethora of available information on ileal EAA losses in broiler chickens, there is a need to further investigate the role of different dietary components (fiber type and viscosity) on ileal EAA losses in broiler chickens and cannulated growing pigs. The hypothesis of this study was that the feed ingredient with the highest WHC will result in a higher ileal EAA flow. Hence, the objective of this study was to determine the effect of CF, WB, and PEC on ileal EAA losses in 26-d old broiler chickens and cannulated growing pigs.

## 2. Materials and Methods

The management of the birds and pigs, experimental procedures, and sample collections for the experiments followed the standard operating procedures for the animal facility as approved by Purdue University Animal Care and Use Committee (protocol numbers 1111000248 and 1311000983).

### 2.1. Feed Ingredients

The analyzed amino acid and proximate contents of the three feed ingredients used in these experiments are reported in Table 1. The WHC of the different ingredients was determined before the diets were mixed. Briefly, 20 g of each of the ground samples was thoroughly mixed in a 200 mL beaker with 100 g of distilled water and was allowed to rest at room temperature for 5 min, after which it was thoroughly mixed again. The contents of each of the beakers were then filtered through a 125 mm Whatman™ filter paper (Cat# 1001 125) for 85 min. The filtrate was collected in a conical flask and weighed at the end of the filtration process. The WHC was calculated as g of water retained/g of sample (Table 1).

### 2.2. Exp. 1: Broiler Chickens

Male Ross 708 broiler chicks were obtained at hatch from a commercial hatchery for this study. All birds were raised in battery cages (Alternative Design Manufacturing, Siloam Spring, AR. Model # SB 4 T) in an environmentally controlled room. On d 0, each bird was individually tagged and group weighed. Birds were individually tagged on day 0 in order to make identification easy on day 21, when birds were individually weighed prior to allotment to treatments. Birds were fed a standard broiler chicken starter diet that met or exceeded the nutrient recommendation [21] from d 0 to 21. On d 21, 576 birds were individually weighed and randomly allocated to nine dietary treatments in a randomized complete block design using the Experimental Animal Allotment Program of [22]. The 576 birds used in the study were selected from more than 600 birds. There were eight blocks/diet and eight birds/cage. Nine semi-purified diets were used in this study with each of the three ingredients—CF, WB, and PEC (Texture Innovation Center, TIC GUMS, Belcamp, MD, USA)—accounting for three diets. The PEC was processed from apple while the CF and WB used in these studies were co-products of corn and wheat, respectively. The significantly lower crude fiber level in the PEC used in these studies may be associated with the processing it has been subjected to. In order to prevent a situation in which birds selectively eat bigger feed particles, an attempt was made to equalize particle size across diets by grinding CF and WB to a size similar to that of PEC using a mill grinder with a 1.0 mm screen. Each of the three test ingredients were added at 100 g/kg diet to diets containing 40, 80, or 120 g casein/kg diet (Table 2). The analyzed amino acid contents of the experimental diets are reported in Table 3.

### 2.3. Expt. 2: Cannulated Pigs

Eighteen growing pigs (Yorkshire × Duroc × Landrace) were fitted with simple T-cannula approximately 15 cm anterior to the ileo–cecal–colonic junction as previously been described [23]. Prior to the current study, the pigs had been used in two earlier studies. The average initial BW of pigs used in this study was 56 ± 0.71 kg. Pigs were allotted to six experimental diets (two fiber types with three levels of casein) resulting in nine pigs per fiber type (three replicates per fiber type). This was repeated twice to give six replicates per fiber type. Each period consisted of 7 days with a 12-h ileal digesta collection on days 6 and 7 after a 5-day adaptation to the experimental diets. Pigs were blocked by body weight, with each block consisting of nine pigs (a total of three blocks per period). Each dietary treatment was repeated once within each block. The ingredient composition of the experimental diets is reported in Table 4 and the analyzed amino acid composition of the diets is reported in Table 5. Diets were offered in two aliquots daily (07:00 h and 19:00 h) at 4% of the body weight of the smallest pig within each block at the start of each period. At the end of the first period, all pigs were fed the standard corn-soybean meal-based diet that was adequate in energy and all nutrients [24] for 7 days (rest period).

### 2.4. Sample Collection

#### 2.4.1. Exp 1

The experimental diets were fed for 5 consecutive days (d 21 to 26). On d 26, birds were weighed individually and euthanized by CO_2_ asphyxiation. Ileal digesta, from the entire ileum, the section between Meckel’s diverticulum and about 5 cm anterior to the ileo–cecal–colonic junction, was flushed into a clean plastic container with distilled water. Digesta within a cage was pooled and stored at −20 °C until they were freeze-dried.

#### 2.4.2. Exp. 2

Ileal digesta samples were collected between 07:00 h and 19:00 h on d 6 and 7 by attaching a plastic bag to the O-ring of the cannula. Each bag was changed frequently at least once every 2 h. To minimize microbial and enzymatic activities post collection, 10 mL of 10% formic acid was added to each bag. Details of sample collection and processing is as described by [25].

### 2.5. Sample Processing

Diets, feed ingredients, and dried ileal digesta samples from pigs were ground to pass through a 0.5-mm screen using a mill grinder (Retsch ZM 100, Retsch GmbH and Co., K.G., Haan, Germany). Ileal digesta from broiler chickens were ground using the coffee grinder. Diets, test feed ingredients, and ileal digesta samples were sent to University of Missouri Experiment Station and Chemical Laboratory for analyses. Corn fiber, WB, and PEC were analyzed for amino acid, crude protein (N × 6.25), moisture, crude fat, crude fiber, ash, acid detergent fiber (ADF), and neutral detergent fiber (NDF, Table 1). Dry matter, titanium, and amino acid contents from the diets and ileal digesta were determined. Duplicate proximate analyses were performed on diets and ileal digesta samples. Dry matter analysis of samples was determined by drying the samples in a drying oven at 105 °C for 16 h (method 934.01, [26]). Nitrogen was determined by the combustion method (TruMac N; Leco Corp., St. Joseph, MI, USA; AOAC, 2000; Method 990.03), with EDTA serving as the internal standard. Samples for amino acid analysis were prepared using a 24-h hydrolysis in 6 *N* hydrochloric acid at 110 °C under an atmosphere of nitrogen. For sulfur-containing amino acids’ (methionine and cysteine) determination, samples were oxidized with performic acid prior to acid hydrolysis. Samples for tryptophan analysis were hydrolyzed using barium hydroxide. Amino acids in hydrolysates were determined by HPLC after postcolumn derivatization. The crude fat contents of CF, WB, and PEC were determined by ether extraction (Method 920.39, [26]). Crude fiber analysis content was determined using AOAC Method 978.10 [26]). Acid detergent fiber was determined using AOAC Method 973.18 (A-D; AOAC Method, [26]), whereas NDF was determined using an Ankom Fiber Analyzer (Ankom Technology, Macedon, NY). Ash contents of CF, WB, and PEC were determined using AOAC Method 942.05 [26]) at the University of Missouri Experiment Station Chemical Laboratory. Titanium concentration in the diets and ileal samples was determined as described by [27].

### 2.6. Calculation and Statistical Analysis

Ileal EAA losses (index method) from birds and pigs on different fiber sources and different levels of casein (nine dietary treatments in Exp. 1 and six dietary treatments in Exp. 2) were calculated as mg of amino acid flow/kg of DM intake (DMI) using the formula previously reported by [14]

Ileal EAA losses (mg/kg of DMI) = (Ti*_i_/Ti_o_)* × *(N_o_)* (1)

where Ti*_i_* represents the dietary titanium concentration in g/kg DMI; *Ti_o_* represents the concentration of titanium in the ileal digesta in g/kg DM output; and *N_o_* represents the ileal digesta nitrogen or amino acid concentration in mg/kg DM.

For each ingredient evaluated in these studies, there were three casein concentrations (40, 80, or 120 g/kg of diet) within each block. Ileal EAA losses were determined from the ordinate intercept, at zero amino acid intake, of the regression of ileal digesta amino acid concentration in mg/kg DMI against dietary amino acid intake in mg/kg DM. Three cages within each block (with three levels: 40, 80, and 120 g of casein/kg diet) served as the experimental unit. This resulted in eight replicate cages of broiler chickens and six individual pigs per treatment. Data were analyzed using the GLM procedure of SAS [28] as a completely randomized block design. Differences in ileal EAA losses between birds fed CF and WB as well as between WB and PEC were compared using contrasts and level of significance was set at 5%.

## 3. Results

The proximate and AA analyses of CF, WB, and PEC are reported in Table 1. With the exception of Leu and Pro, WB had higher amino acids and total amino acid contents than CF (total amino acid 14.0 vs. 9.8%) which, in turn, had higher amino acid contents than PEC (WB > CF > PEC). Of the three samples evaluated, WB had the highest crude protein content (16.5%), while the crude protein contents of CF and PEC were similar (~10.8%). Unlike CF and WB, whose amino acid profiles (CF total amino acid = 9.8% and WB total amino acid = 14.0%) represent 91 and 85% of their respective crude protein contents, the total amino acid of PEC was 1.1%, which was approximately 10% of its crude protein contents (Table 1). The crude fiber content of PEC (0.19%) was about 1.9% of that of CF and WB. Additionally, ADF and NDF contents of PEC (0.06 and 0.00%, respectively) were the lowest of the three samples, while CF had the lowest (0.8%) mineral contents, as reflected by its ash content. Wheat bran had the highest mineral content (6.3%). The respective WHC for CF, WB, and PEC were 2.9, 3.1, and >5.0 g of water/g of sample (Table 1).

### 3.1. Ileal Endogenous Amino Acid Losses (Broiler Chicken)

#### Exp. 1

The effect of CF, WB, and PEC on ileal EAA losses in 26-day old broiler chickens is presented in Table 6. Ileal endogenous losses of His, Glu, and Pro in birds fed CF-based diets was higher (*p* < 0.05) compared with birds on PEC-based diets (Table 6). Birds fed diets containing CF showed a tendency of higher (*p* < 0.10) endogenous losses of Ile, Met, nitrogen and total amino acid compared with birds fed the PEC diet. The nitrogen and total amino acid losses in birds fed the PEC-based diet was 61 and 66%, respectively, of losses in birds fed CF-based diets. Contrasting ileal EAA losses between birds fed the CF- and WB-based diets showed higher (*p* < 0.05) EAA losses for nitrogen, total amino acid, and six amino acids (His, Ile, Met, Glu, Pro, and Tyr) and a tendency towards higher (*p* < 0.10) EAA losses for Leu, Phe, Thr, Val, Asp, and Ser compared with birds fed a diet containing WB. Contrasts of EAA losses between birds fed the WB- and PEC-based diets showed higher (*p* < 0.05) losses for His, Glu, and Pro, while Ile, Met, Asp, Tyr, and total amino acid showed a tendency of higher (*p* < 0.10) losses in birds fed diets containing WB.

### 3.2. Ileal Endogenous Amino Acid Losses (Pig)

#### Exp. 2

The effect of CF and PEC on ileal EAA losses in cannulated pigs is presented in Table 7. Corn fiber resulted in higher (*p* < 0.05) ileal endogenous His, Leu (4.6%), and Tyr losses. Isoleucine, Phe, Thr, and Cys losses showed a tendency to increase with CF.

## 4. Discussion

The objective of this study was to investigate the effect of different test feed ingredient (WB, CF, and PEC) on ileal EAA losses in broiler chickens and cannulated pigs using the regression method. The level of fiber in CF and WB was high compared to that of PEC. In nonruminant animals, fiber, especially cellulose, is regarded as an antinutritional factor because of the inability of the animal to effectively utilize fiber due to their inability to digest it. At the same time, however, dietary fiber could affect the health of the GIT, especially the hindgut. Fiber in feed ingredients have generally been classified as soluble (ability to absorb moisture) or insoluble (resistant to moisture absorption). In general, fiber influences digesta passage rate and, as in the case of fiber with high WHC, the bulkiness of the digesta could result in an increase in the size (or volume) of the GIT and this could lead to an increase in the level of interaction between the digesta and the wall of the GIT as the digesta travels along the GIT. Corn fiber is a classic example of insoluble fiber with a low WHC (2.9 g of water retained/g of sample), while the PEC used in this study was soluble with relatively high WHC (>5.0 g/g). Based on our observation, the WHC of the WB (3.1 g/g) used in this study was higher than that of CF (2.9 g/g) but lower than that of PEC (<5.0 g/g). The effect of dietary fiber on feed intake, digesta passage rate, gut health, and EAA losses in poultry and swine has been well-documented [18,19,29,30]. The characteristics of the feed ingredient fiber that may influence EAA flow include its potential for bulkiness, which may result in abrasion and adsorption as well as its tendency for high viscosity arising from the potential for high WHC, especially for soluble fiber. The significance of the nature of dietary fiber (soluble or insoluble) on ileal EAA losses has not been extensively documented. Available information [9] shows that soluble fiber is more likely to result in increased ileal EAA losses, while insoluble fiber (cellulose such as solkafloc) may not [9,10]. Results from the current study, however, could not be explained solely from the perspective of high viscosity, dietary crude protein level, and dietary crude fiber contents.

Increasing level of dietary nitrogen, protein, peptides, and amino acids have been shown to increase basal ileal EAA losses in non-ruminant animals [1,14,31,32]. The crude protein levels of the different ingredients evaluated in the current study were 10.8, 16.5, and 10.8%, respectively, for CF, WB, and PEC. However, a substantial quantity of the nitrogen in the PEC was not from protein origin compared with the remaining two ingredients (CF and WB). For instance, only about 10% of the nitrogen in the PEC was from amino acids (protein), which means about 90% of the nitrogen in the PEC was from non-protein source(s). The respective values for CF and WB were 91 and 85%. Furthermore, the analyzed fiber content in the PEC was about 2% of what it was in CF and WB. The ADF value for PEC was about 0.5% of that of CF and WB and the NDF was not detected in the PEC. Based on this, while CF and WB have similar chemical compositions, the chemical composition of PEC was different.

The higher ileal His, Glu, and Pro and the tendency for higher nitrogen, total amino acid, Ile, and Met losses in broiler chickens fed diets containing CF and WB compared with those fed diets containing PEC could be attributed to either the higher level of amino acids, crude fiber or both in the CF and WB diets. It has been reported in broiler chickens that higher dietary fiber level could result in higher ileal EAA losses [17], while [10] reported no difference in ileal endogenous total amino acid losses in the non-challenged broiler chickens fed low and high dietary fiber levels. The former study [17] employed complete diets with fiber from corn, wheat, soybean meal, and cellulose while the later study [10] used semi-purified diets with cellulose as the only source of fiber. The differences in the results of the two studies could be attributed to the type and level of fiber used. Even for the amino acids that showed no significant effect of ingredient type, the EAA losses from broiler chickens fed the PEC diets were consistently lower (PEC values for nitrogen, threonine, and total amino acid were 64, 76, and 68% of the average values for CF and WB). It is important to note that when values from birds fed CF were compared with values from birds fed WB, birds fed CF had higher ileal endogenous nitrogen, total amino acid, His, Ile, Met, Glu, Pro, and Tyr with six other amino acids showing tendency for higher losses. This finding is interesting based on the fact that both fiber types had similar crude fiber (10.0 vs. 9.4%) and ADF (11.3 vs. 11.4%) levels. Crude protein (10.8 for CF vs. 16.5% for WB), ash (0.8 vs. 6.3%), crude fat (5.6 vs. 3.5%), and NDF (49.8 vs. 44.4%) were the only components of the two ingredients that were different. Based on the available information on the effect of dietary amino acid peptides on ileal EAA flow, the additional dietary amino acids coming from WB were expected to have resulted in a higher level of EAA flow for birds on WB-based diets compared with birds on CF-based diets (9.4-percentage point difference for nitrogen). More importantly, WB contained a relatively higher level of nitrogen coming from non-protein source(s) compared with CF (15 vs. 9%). Hence, it is likely that the higher EAA and nitrogen losses from the CF-fed birds could not be explained by its crude protein level alone.

Seven amino acids and total amino acid showed higher (or tendency to be higher) endogenous losses when diet containing WB was fed to 26-d old broiler chickens compared with the PEC diet. This difference in EAA losses becomes even greater when losses from birds fed diets containing CF are compared with flow from birds fed PEC diets. This observation could be attributed to differences in the crude protein (especially the proportion of nitrogen from protein) and crude fiber levels of the two ingredients. The high level of nitrogen from non-protein sources in PEC resulted in lower basal ileal EAA losses. The effect of high viscosity of the digesta as a result of feeding PEC-containing diets was not sufficient to offset the effect of the quantity of nitrogen from non-protein sources on ileal EAA losses in 26-d old broilers.

The effect of CF on ileal EAA losses in cannulated growing pigs was different (higher) in only three amino acids (His, Leu, and Tyr) with similar endogenous nitrogen (4192 vs. 3997 kcal/kg DMI) and total amino acid (21,971 vs. 20,280 mg/kg DMI) values. Unlike in broilers, these data suggest that the nitrogen from PEC which is mainly from non-protein source(s) had a much higher effect on ileal EAA losses compared with what was observed in the broiler chickens. One of the questions that needs to be addressed is the possibility that the presence of nitrogen from non-protein nitrogen sources could have served as the nutrients for microbes in the distal section of the small intestine, the ileum, and hence might have resulted in the growth and proliferation of these microbes, leading to an increase in ileal EAA losses. This becomes a possibility because ileal endogenous nitrogen loss was 39% higher in broiler chickens fed CF compared with those fed PEC. However, in the cannulated pigs, the increase was less than 7%. The difference in these values (CF vs. PEC) in the pig was definitely associated with the interaction of PEC with the gastrointestinal tract (e.g., mucin, microbes). Ileal digesta bacteria contents have been shown to be influenced by the viscosity of the diet [33] and dietary fiber types [34]. The level of *Lactobacillus* bacteria has been shown to be significantly increased by pea fiber compared with the control and soybean fiber [35]. Furthermore, soybean fiber significantly increased the level of *E. coli* in the ileal digesta compared with wheat bran. The implication for this study is unclear because the levels of non-protein nitrogen in the different diets used were not measured.

Another factor that could be considered is the effect of dietary fiber on gizzard development and its implications on ileal EAA losses. Although it has been established that high levels of dietary insoluble fiber could result in a reduction in the length of the small intestine [36,37] as well as an increase in gizzard weight [37,38,39,40], the effects of this on gizzard weight are not expected to influence ileal EAA losses in this study for a number of reasons. First, the level of fiber in the diets used in this study was less than 1% of the diet. Secondly, the particle size of all the ingredients used in the current study were similar, as already discussed. Thirdly, irrespective of what goes on within the gizzard, this organ is not known to produce any secretions that could influence the digestive capacity of the birds. More research needs to be conducted to further understand the effect of dietary insoluble fiber level on ileal EAA losses, as it related to the length of the mid gut.

This study showed that the effect of WHC on ileal EAA losses may not be as significant as the influence of crude fiber type and concentration. However, between CF and WB, it may be concluded that the effect of crude protein or total amino acid may not be as significant in ileal EAA losses as that of NDF. Finally, CF, the least soluble among the three samples evaluated in this study resulted in significantly higher EAA losses in 26-d old broilers compared with WB, which had higher crude protein and total amino acid concentration, and PEC, which had the highest WHC but lowest total amino acid and crude fiber. In order to fully understand the results from this study, it is important to estimate the effect of different source of nitrogen (protein nitrogen and non-protein nitrogen) on basal ileal EAA losses in broiler chickens and cannulated pigs. Furthermore, quantification of the type and relative quantities of microbes in the ileal digesta could answer some of the questions that arose from these studies.

## 5. Conclusions

The results from these studies showed that the corn fiber resulted in similar ileal endogenous amino acid losses in both broiler chickens and cannulated growing pigs. Furthermore, high water-holding capacity did not translate into a high level of endogenous nitrogen and amino acid losses in both species. However, the source of dietary nitrogen (protein vs. non-protein nitrogen) may have a significant effect on basal ileal endogenous nitrogen and amino acid losses and the level of losses may differ depending on animal species.

## Figures and Tables

**Table 1:** Analyzed amino acid contents and proximate analysis of corn fiber, wheat bran, and pectin.

	Corn Fiber	Wheat Bran	Pectin ^1^
Indispensable amino acid, %			
Arg	0.41	1.11	0.06
His	0.36	0.44	0.03
Ile	0.34	0.51	0.05
Leu	1.19	0.97	0.08
Lys	0.30	0.63	0.06
Met	0.17	0.23	0.01
Phe	0.48	0.62	0.05
Thr	0.38	0.48	0.04
Try	0.07	0.18	<0.04
Val	0.49	0.76	0.06
Dispensable amino acid, %			
Ala	0.68	0.74	0.05
Asp	0.55	1.12	0.12
Cys	0.23	0.33	0.01
Glu	1.79	2.92	0.16
Gly	0.42	0.85	0.05
Pro	1.01	0.95	0.05
Ser	0.42	0.56	0.04
Tyr	0.33	0.41	0.03
Total amino acid	9.81	13.96	1.07
Proximate analyses, %			
Crude protein ^2^	10.78	16.47	10.75
Moisture	8.25	9.66	10.05
Crude fat	5.56	3.48	0.60
Crude fiber	9.98	9.43	0.19
Ash	0.75	6.34	2.43
Acid detergent fiber	11.25	11.42	0.06
Neutral detergent fiber	49.84	44.41	0.00
Water-holding capacity, g/g ^3^	2.90	3.01	>5.00

^1^ Pectin LM 32 Powder from Texture Innovative Center (TIC Gum), White Marsh MD 21162, USA. ^2^ Crude protein = Nitrogen × 6.25. ^3^ Grams of water retained/gram of sample.

**Table 2:** Ingredient composition of experimental diets fed to broiler chickens (on as-is basis; Exp. 1).

Ingredient:		Corn Fiber				Wheat Bran				Pectin ^1^		
Casein, g/kg:		40	80	120		40	80	120		40	80	120
Ingredient g/kg												
Corn starch		85.8	44.3	2.8		85.8	44.3	2.8		85.8	44.3	2.8
Dextrose		640	640	640		640	640	640		640	640	640
Casein ^2^		40	80	120		40	80	120		40	80	120
Corn fiber		100	100	100		0	0	0		0	0	0
Wheat bran		0	0	0		100	100	100		0	0	0
Pectin		0	0	0		0	0	0		100	100	100
Soy oil		50	50	50		50	50	50		50	50	50
Vitamin-mineral												
premix ^3^		5	5	5		5	5	5		5	5	5
Monocalcium phosphate		17	17	17		17	17	17		17	17	17
NaHCO_3_		12	12	12		12	12	12		12	12	12
KCl		4	4	4		4	4	4		4	4	4
MgO		0.7	0.7	0.7		0.7	0.7	0.7		0.7	0.7	0.7
Choline chloride		2.5	2.5	2.5		2.5	2.5	2.5		2.5	2.5	2.5
Limestone		18	19.5	21		18	19.5	21		18	19.5	21
Titanium dioxide premix ^4^		25	25	25		25	25	25		25	25	25
Total		1000	1000	1000		1000	1000	1000		1000	1000	1000
Calculated nutrients and energy												
Crude protein (N × 6.25), g/kg		44.4	78.4	112.4		49.7	83.7	117.7		44.2	78.2	112.2
ME, kcal/kg		3334	3358	3381		3464	3488	3511		3674	3698	3721
Calcium, g/kg		9.9	10.7	11.5		10.0	10.8	11.7		10.0	10.8	11.6
Phosphorus, g/kg		4.2	4.4	4.6		5.3	5.6	5.8		4.2	4.4	4.6
Non-phytate P, g/kg		3.9	4.3	4.6		4.1	4.5	4.8		3.9	4.3	4.6
Ca:P		2.4	2.4	2.5		1.9	1.9	2.0		2.4	2.5	2.5

^1^ Pectin LM 32 Powder from Texture Innovative Center (TIC GUMS), White Marsh MD 21162, USA; ^2^ Casein; ^3^ Provided per kg of diet: iron, 71.6 mg; copper, 11.0 mg; manganese, 178.7 mg; zinc, 178.7 mg; iodine, 3.0 mg; selenium, 0.4 mg. vitamin A (retinyl acetate), 18,904.3 IU; vitamin D_3_ (cholecalciferol), 9480.0 IU; vitamin E (DL-α-tocopheryl acetate), 63.0 IU; vitamin K activity, 6.4 mg; thiamine, 3.2 mg; riboflavin, 9.4 mg; pantothenic acid, 34.7 mg; niacin, 126.0 mg; pyridoxine, 4.7 mg; folic acid, 1.6 mg; biotin, 0.5 mg; vitamin B_12_, 35.4 mcg; choline, 956.9 mg; ^4^ Prepared by mixing 1 g of titanium dioxide with 4 g of corn starch.

**Table 3:** Analyzed dietary amino acid contents of the diets fed to broiler chickens (as fed basis; Exp. 1).

Ingredient:		Corn Fiber				Wheat Bran				Pectin ^1^		
Casein, g/kg:		40	80	120		40	80	120		40	80	120
Dry matter, %		91.6	91.5	91.3		91.4	91.3	91.3		91.2	91.4	91.4
Nitrogen, %		0.69	1.21	1.63		0.72	1.31	2.04		0.60	1.20	1.70
Indispensable amino acid, %												
Arg		0.14	0.25	0.35		0.19	0.32	0.40		0.09	0.19	0.32
His		0.13	0.22	0.32		0.12	0.23	0.31		0.08	0.18	0.29
Ile		0.24	0.42	0.63		0.21	0.43	0.59		0.18	0.37	0.61
Leu		0.47	0.80	1.16		0.41	0.82	1.09		0.31	0.65	1.06
Lys		0.31	0.57	0.86		0.30	0.63	0.85		0.25	0.52	0.85
Met		0.11	0.20	0.32		0.11	0.23	0.30		0.09	0.19	0.29
Phe		0.23	0.40	0.58		0.22	0.43	0.58		0.17	0.34	0.55
Thr		0.19	0.34	0.50		0.20	0.39	0.51		0.15	0.29	0.48
Try		0.05	0.10	0.13		0.06	0.09	0.16		0.05	0.09	0.13
Val		0.29	0.51	0.77		0.27	0.54	0.73		0.21	0.45	0.73
Dispensable amino acid, %												
Ala		017	0.27	0.38		0.17	0.29	0.37		0.10	0.20	0.32
Asp		0.29	0.52	0.76		0.32	0.60	0.78		0.22	0.45	0.73
Cys		0.03	0.05	0.07		0.04	0.07	0.08		0.02	0.03	0.05
Glu		0.96	1.71	2.52		1.00	1.94	2.54		0.70	1.47	2.41
Gly		0.11	0.18	0.24		0.14	0.22	0.27		0.07	0.13	0.21
Pro		0.49	0.83	1.23		0.43	0.89	1.16		0.34	0.72	1.15
Ser		0.21	0.38	0.54		0.23	0.46	0.60		0.16	0.31	0.53
Tyr		0.16	0.31	0.49		0.16	0.35	0.51		0.12	0.26	0.46
Total amino acid		4.67	8.13	11.93		4.66	9.03	11.85		3.36	6.89	11.23

^1^ Pectin LM 32 Powder from Texture Innovative Center (TIC Gum), White Marsh MD 21162, USA.

**Table 4:** Composition of the experimental diets fed to pigs, g/kg (on as-fed basis; Exp. 2).

Ingredient:		Corn Fiber				Pectin		
Casein,g/kg:		40	80	120		40	80	120
Ingredient, g/kg								
Corn starch		87.8	46.3	4.8		87.8	46.3	4.8
Dextrose		640	640	640		640	640	640
Casein		40	80	120		40	80	120
Corn fiber		100	100	100		0	0	0
Pectin		0	0	0		100	100	100
Soy oil		50	50	50		50	50	50
Monocalcium phosphate ^1^		17	17	17		17	17	17
NaHCO_3_		12	12	12		12	12	12
KCl		4.00	4.00	4.00		4.00	4.00	4.00
MgO		0.7	0.7	0.7		0.7	0.7	0.7
Choline chloride		2.5	2.5	2.5		2.5	2.5	2.5
Limestone		18	19.5	21		18	19.5	21
Vitamin ^2^		1.5	1.5	1.5		1.5	1.5	1.5
Mineral ^3^		1.0	1.0	1.0		1.0	1.0	1.0
Selenium premix ^4^		0.5	0.5	0.5		0.5	0.5	0.5
Titanium dioxide premix ^5^		25	25	25		25	25	25
Total		1000	1000	1000		1000	1000	1000
Calculated nutrients and energy								
Calcium, g/kg		9.9	10.7	11.5		10.0	10.8	11.6
Phosphorus, g/kg		4.2	4.4	4.6		4.2	4.4	4.6
Non-phytate phosphorus, g/kg		3.9	4.3	4.6		3.9	4.3	4.6
Ca:tP		2.3	2.4	2.5		2.4	2.4	2.5

^1^ 160g of calcium and 210 g pf phosphorus per kilogram. ^2^ Vitamin premix supplied per kilogram of diet: 3635 IU vitamin A, 363 IU vitamin D3, 26.4 IU vitamin E, 3.6 mg vitamin K, 1206 μg menadione, 21.2 μg vitamin B12, 4.2 mg riboflavin, 13.5 mg d-pantothenic acid, and 19.5 mg niacin. ^3^ Mineral premix supplied per kilogram diet: 9 mg Cu (as copper sulfate), 0.34 mg I (as Ca iodate), 97 mg Fe (as ferrous sulfate), Fe (as ferrous sulfate), 12 mg Fe (as ferrous sulfate), and 97 mg Zn (as zinc oxide). ^4^ Provided 600 μg of Se (as sodium selenite) per gram of premix. ^5^ Prepared by mixing 1 g of titanium dioxide with 4 g of cornstarch.

**Table 5:** Analyzed amino acid contents of the experimental diets fed to pigs (Exp. 2; on as-fed basis).

Ingredient:		Corn Fiber				Pectin		
Casein, g/kg:		40	80	120		40	80	120
Nitrogen		0.78	1.46	1.82		0.70	1.32	1.86
Indispensable amino acid, %								
Arg		0.15	0.28	0.36		0.12	0.22	0.32
His		0.14	0.25	0.33		0.10	0.18	0.29
Ile		0.24	0.48	0.64		0.22	0.41	0.60
Leu		0.48	0.90	1.19		0.38	0.68	1.05
Lys		0.36	0.68	0.91		0.34	0.59	0.88
Met		0.13	0.24	0.34		0.12	0.20	0.30
Phe		0.25	0.45	0.60		0.20	0.37	0.54
Thr		0.21	0.39	0.52		0.18	0.30	0.48
Try		0.06	0.12	0.16		0.07	0.12	0.16
Val		0.39	0.66	0.84		0.37	0.54	0.77
Dispensable amino acid, %								
Ala		0.19	0.32	0.41		0.14	0.22	0.33
Asp		0.33	0.61	0.81		0.29	0.50	0.75
Cys		0.04	0.06	0.06		0.02	0.03	0.04
Glu		1.07	1.93	2.54		0.93	1.52	2.32
Gly		0.13	0.21	0.26		0.10	0.16	0.22
Pro		0.51	0.98	1.31		0.43	0.98	1.18
Ser		0.23	0.42	0.56		0.19	0.33	0.52
Tyr		0.20	0.39	0.53		0.17	0.34	0.47
Total amino acid		5.13	9.40	12.39		4.44	7.91	11.29

**Table 6:** Ileal endogenous losses (mg/kg DMI) in 26 d-old broiler chickens fed three different fiber types using the regression method (Exp. 1).

Ingredient:	Corn Fiber	Wheat Bran	Pectin ^1^	SEM ^2^	*p*-Value	Contrasts ^3^	
	Endogenous Losses, mg/kg DMI					CF vs. WB	WB vs. PEC
Nitrogen	4088	3704	2504	469.1	0.089	0.038	0.101
Indispensable amino acid							
Arg	1118	1225	798	173.7	0.243	0.222	0.113
His	558 ^a^	531 ^a^	300 ^b^	58.9	0.021	0.011	0.020
Ile	1109	989	695	109.1	0.059	0.023	0.086
Leu	1693	1500	1057	205.3	0.129	0.053	0.158
Lys	1442	1224	947	212.7	0.299	0.130	0.378
Met	342	315	161	50.0	0.058	0.028	0.054
Phe	889	837	578	116.9	0.182	0.089	0.149
Thr	1249	1155	908	128.8	0.205	0.091	0.205
Try	197	150	169	33.3	0.621	0.567	0.695
Val	1395	1356	970	154.7	0.150	0.081	0.108
Dispensable amino acid							
Ala	1049	1036	759	139.2	0.295	0.172	0.189
Asp	1803	1711	1127	224.5	0.118	0.059	0.096
Cys	557	416	381	76.1	0.272	0.135	0.758
Glu	3054 ^a^	2788 ^ab^	1632 ^b^	338.0	0.031	0.014	0.036
Gly	1102	1067	758	134.4	0.189	0.101	0.136
Pro	1306 ^a^	1279 ^a^	803 ^b^	98.9	0.008	0.005	0.007
Ser	1336	1224	896	162.3	0.188	0.084	0.184
Tyr	711	664	459	78.8	0.103	0.048	0.096
Total amino acid	22,387	20,761	14,717	2343.2	0.097	0.043	0.098

^1^ Pectin LM 32 Powder from Texture Innovative Center (TIC Gum), White Marsh MD 21162, USA. ^2^ Standard error of the mean. ^3^ CF = Corn fiber; WB = wheat bran; PEC = Pectin. ^ab^ Means within a row without a common superscript differ (*p* < 0.05).

**Table 7:** The effect of corn fiber and pectin on ileal endogenous amino acid losses (mg/kg DMI) in cannulated growing pigs (Exp. 2).

	Corn Fiber	Pectin	*p*-Value	SD ^1^
Nitrogen	4192	3997	0.860	2296
Indispensable amino acid				
Arg	987	861	0.701	686
His	427	234	0.019	156
Ile	610	380	0.089	269
Leu	1106	640	0.042	447
Lys	783	517	0.135	359
Met	157	97	0.156	83
Phe	613	361	0.061	265
Thr	1106	694	0.090	483
Try	184	153	0.353	70
Val	779	562	0.225	364
Average	675	450		
Dispensable amino acid				
Ala	937	774	0.502	504
Asp	1381	948	0.128	572
Cys	411	198	0.084	220
Glu	1858	1292	0.177	851
Gly	2349	2816	0.630	1954
Pro	7909	8420	0.905	8612
Ser	1104	770	0.219	552
Tyr	479	285	0.049	193
Average	2054	1938		
Total amino acid	21,971	20,280	0.8005	13,961

^1^ Standard deviation.
